# Comprehensive Overview on the Chemistry and Biological Activities of Selected Alkaloid Producing Marine-Derived Fungi as a Valuable Reservoir of Drug Entities

**DOI:** 10.3390/biomedicines9050485

**Published:** 2021-04-28

**Authors:** Fadia S. Youssef, Jesus Simal-Gandara

**Affiliations:** 1 Department of Pharmacognosy, Faculty of Pharmacy, Ain Shams University, Cairo 11566, Egypt; fadiayoussef@pharma.asu.edu.eg; 2 Nutrition and Bromatology Group, Department of Analytical and Food Chemistry, Faculty of Food Science and Technology, Ourense Campus, University of Vigo, E32004 Ourense, Spain

**Keywords:** alkaloids, biology, chemistry, fungi, marine

## Abstract

Marine-associated fungal strains act as a valuable reservoir of bioactive diverse secondary metabolites including alkaloids which are highly popular by their biological activities. This review highlighted the chemistry and biology of alkaloids isolated from twenty-six fungal genera associated with marine organisms and marine sea sediments. The selected fungi are from different marine sources without focusing on mangroves. The studied fungal genera comprises *Acrostalagmus*, *Arthrinium*, *Chaetomium*, *Cladosporium*, *Coniothyrium*, *Curvularia*, *Dichotomomyces*, *Eurotium*, *Eutypella*, *Exophiala*, *Fusarium*, *Hypocrea*, *Microsphaeropsis*, *Microsporum*, *Neosartorya*, *Nigrospora*, *Paecilomyces*, *Penicillium*, *Pleosporales*, *Pseudallescheria, Scedosporium*, *Scopulariopsis*, *Stagonosporopsis*, *Thielavia*, *Westerdykella,* and *Xylariaceae.* Around 347 alkaloid metabolites were isolated and identified via chromatographic and spectroscopic techniques comprising 1D and 2D NMR (one and two dimensional nuclear magnetic resonance) which were further confirmed using HR-MS (high resolution mass spectrometry) and Mosher reactions for additional ascertaining of the stereochemistry. About 150 alkaloids showed considerable effect with respect to the tested activities. Most of the reported bioactive alkaloids showed considerable biological activities mainly cytotoxic followed by antibacterial, antifungal, antiviral, antioxidant; however, a few showed anti-inflammatory and antifouling activities. However, the rest of the compounds showed weak or no activity toward the tested biological activities and required further investigations for additional biological activities. Thus, alkaloids isolated from marine-associated fungi can afford an endless source of new drug entities that could serve as leads for drug discovery combating many human ailments.

## 1. Introduction

Nowadays fungi isolated from marine resources serve as promising tools for the alleviation of a large number of hazardous diseases that adversely affect human health such as bacterial and viral infections as well as cancers [1,2]. These prominent effects are greatly relied upon their richness by large categories of secondary metabolites represented by peptides, steroids, terpenoids, lactones, and alkaloids [3,4].These activities are mainly anti-inflammatory, antibacterial, anticancer, and antiviral activities [5,6]. A vast variation in the function and structure of the abundant metabolites in the marine-derived fungal strains is undoubtedly based upon the considerable diversity in the environment where these organisms exist regarding its chemical and physical formation [7].

In addition, these fungal metabolites showed an acceptable oral-bioavailability and physico-chemical manner offering a safer biomedical alternative relative to synthetic entities that constitute a crucial importance in the process of formulation of various dosage forms [8,9]. Besides, plethora of alkaloids obtained from marine fungi exhibited a wide range of biological effectiveness [10,11,12,13].

Thus, this review aimed to comprehensively explore the diverse alkaloids isolated and identified from nearly most of the fungal strains belonging to diverse genera associated with different marine organisms and sediments regarding their chemistry and biology. The selected fungi are from different marine sources without focusing on mangroves. These include *Acrostalagmus*, *Arthrinium*, *Aspergillus*, *Chaetomium*, *Cladosporium*, *Coniothyrium*, *Curvularia*, *Eurotium*, *Eutypella*, *Exophiala*, *Fusarium*, *Hypocrea*, *Microsphaeropsis*, *Microsporum*, *Neosartorya*, *Nigrospora*, *Paecilomyces*, *Penicillium*, *Pleosporales*, *Pseudallescheria*, *Scedosporium*, *Scopulariopsis*, *Stagonosporopsis*, *Thielavia*, *Westerdykella,* and *Xylariaceae.* The fungal genera were classified in an alphabetical order where 347 alkaloid compounds were reported. The majority of the reported alkaloids revealed antioxidant, cytotoxic, antiviral, antifungal, antibacterial, and anti-inflammatory as well as antifouling activities. Relevant data of the mentioned fungal strains were collected till December 2020 from different databases including Scifinder (https://scifinder.cas.org/scifinder/login, accessed on 1 December 2020) for studies about chemical constituents; however, for biology-related researches, data were gathered from both PubMed (http://www.ncbi.nlm.nih.gov/pubmed/, accessed on 1 December 2020) as well as Web of Knowledge (http://www.webofknowledge.com, accessed on 1 December 2020).

## 2. Classification of Different Classes of Alkaloids According to the Diverse Genera of the Marine-Associated Fungi in an Alphabetical Order

### 2.1. Acrostalagmus

**Figure 1:**
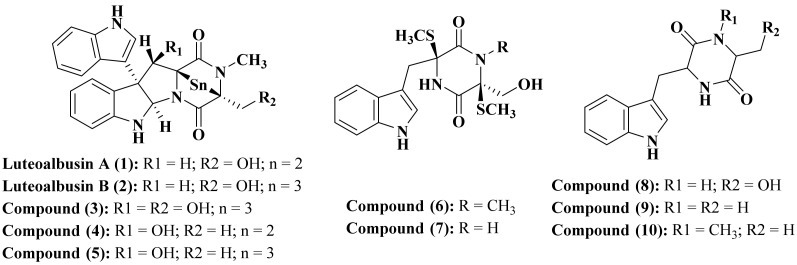
Alkaloids isolated from *Acrostalagmus* species.

Luteoalbusins A (1) and B (2) are two new alkaloids of indole diketopiperazine type which were isolated from *Acrostalagmus luteoalbus* obtained from deep-sea marine sediments existing in the South China Sea in addition to eight previously isolated alkaloids (3–10) as illustrated in Figure 1. Compounds (1–5) exhibited a potent cytotoxic activity against HepG-2, MCF-7, SF-268, as well as NCI-H460 cell lines. Noteworthy to mention that luteoalbusins A (1) and B (2) showed superior cytotoxic activity comparable to other isolated compounds and to cisplatin showing IC_50_ ranging between 0.23 and 1.29 μM [14].

### 2.2. Arthrinium

**Figure 2:**
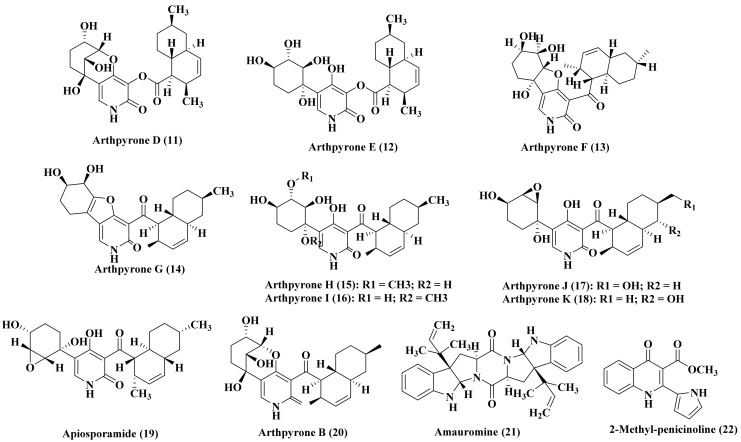
Alkaloids isolated from *Arthrinium* and *Auxarthron* species.

Arthpyrones D–K (11–18), new biologically active hydroxy pyridone alkaloids were isolated and structurally elucidated from *Arthrinium* sp., a fungal strain associated with a marine sediment sample collected from the South China Sea, together with other previously isolated alkaloids known as apiosporamide (19) and arthpyrone B (20) (Figure 2). Arthpyrones F–I (13–16) and apiosporamide (19) exhibited significant antimicrobial activity against *Staphylococcus aureus* and *Mycobacterium smegmatis* with IC_50_ values ranging from 1.66 to 42.8 μM. Apiosporamide (19) revealed an observable cytotoxic activity versus U2OS and MG63, two osteosarcoma cancer cells of human origin, displaying 19.3 and 11.7 μM, respectively as IC_50_ values. However none of the tested samples revealed any acetyl choline esterase inhibitory activity [15].

### 2.3. Auxarthron

Two alkaloids, amauromine (21) of diketopiperazine type and 2-methyl-penicinoline (22) of quinolinone type were isolated from the marine sponge *Ircinia variabilis* associated *Auxarthron reticulatum* fungus. Amauromine (21) acts as a potent antagonist to CB1 receptor showing a strong selective binding to human cannabinoid CB1 receptor with Ki = 178 nM and Kb equals to 66.6 nM as evaluated in cAMP assays however 2-methyl-penicinoline (22) showed very mild binding potential to CB receptors (Figure 2) [16].

### 2.4. Chaetomium

**Figure 3:**
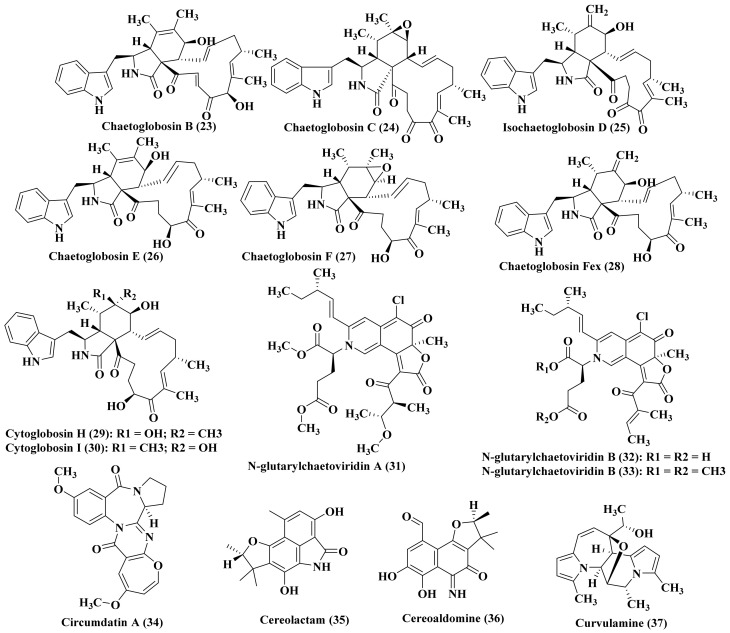
Alkaloids isolated from *Chaetomium*, *Cladosporium*, *Coniothyrium,* and *Curvularia* species.

In depth phytochemical investigation of *Chaetomium globosum* derived from a deep- sea marine sediment from the Indian oceans led to the isolation of a series of cytoglobosins comprising chaetoglobosin B (23), chaetoglobosin C (24), isochaetoglobosin D (25), chaetoglobosin E (26), chaetoglobosin F (27), chaetoglobosinFex (28) in addition to the new alkaloids, cytoglobosins H (29) and I (30). In the evaluation of the cytotoxicity of the previously mentioned compounds on MDA-MB-231, B16F10, and LNCaP, only chaetoglobosin E (26) exhibited a pronounced antiproliferative activity via induction of apoptosis on LNCaP as well as B16F10 cancer cells displaying IC_50_ of 0.62 and 2.78 µM, respectively [17]. Noteworthy to highlight that chaetoglobosin Fex (28) showed a wide array of biological activities showing an immunosuppressive effect thus it could serve as a good candidate in the treatment of autoimmune inflammatory disorder as it effectively inhibited the stimulation of bone marrow-derived dendritic cells which was induced by poly(I:C). This was achieved via attenuation of the production of IFN-β in both mRNA as well as protein level in addition to inhibiting the phosphorylation of IκB-α, IRF-3, p38, and JNK, exerting no effect on ERK1/2 for p38 and JNK. [18]. *Chaetomium globosum*, marine-derived endophytic fungus, also prohibits the stimulation of the inflammatory mediators through Toll-like receptor 4 signaling present in macrophages. Pre-incubation of LPS-stimulated macrophages cells with chaetoglobosin Fex (28) (0.5 mg/mL) significantly inhibited LPS-induced intracellular TNF-α production (15.2% inhibition by 0.5 mg/mL, 21.3% inhibition by 1 mg/mL, and 56.7% inhibition by 2 mg/mL). Treatment with different concentration of chaetoglobosin Fex (28) also blocked IL-6 secretion induced by LPS for 20 h [19]. Additionally, three azaphilone alkaloids possessing glutamine moieties were newly isolated from the deep sea sediment associated *Chaetomium globosum* which are N-glutarylchaetoviridins A–C (31–33). N-glutarylchaetoviridin C (33) exerted a significant activity versus human gastric cancer cell line (MGC-803) and human ovarian cancer cell line (HO8910) displaying IC_50_ values equal to 6.6 and 9.7 µM, respectively (Figure 3) [20].

### 2.5. Cladosporium

In depth phytochemical investigation of the mycelium extract of the marine-derived fungus *Cladosporium*, fungal strain isolated from the surface of the marine red alga *Chondria crassicualis* collected at Yokji Island, Kyeongnam Province, Korea, resulted in the isolation of benzodiazepine alkaloid, circumdatin A (34). It shows no antibacterial activity versus methicillin-resistant *S. aureus*, *S. aureus,* or multidrug-resistant *S. aureus*. However, it showed a powerful antioxidant activity evidenced by its UV-A protection potential displaying ED_50_ = 82.0 µM which in turn highlights its superior activity comparable to oxybenzone, positive control widely used in sunscreen formulation, that showed ED_50_ = 350 µM (Figure 3) [21].

### 2.6. Coniothyrium

Two polyketide skeleton bearing alkaloids namely, (−)-cereolactam (35) and (−)-cereoaldomine (36) were isolated from *Coniothyrium cereal*, a marine-derived fungus which was isolated from the Baltic sea alga *Enteromorpha* sp. (Figure 3). They selectively inhibit human leukocyte elastase with 9.28 and 3.01 μM, respectively as IC_50_ values [22].

### 2.7. Curvularia

*Curvularia*, the marine-associated fungus, was isolated from the gut of *Argyrosomus argentatus* collected from the Yellow Sea yielded the isolation of an alkaloid of a novel skeleton that is termed curvulamine (37) that showed a potent antimicrobial activity (Figure 3) [23].

### 2.8. Dichotomomyces

**Figure 4:**
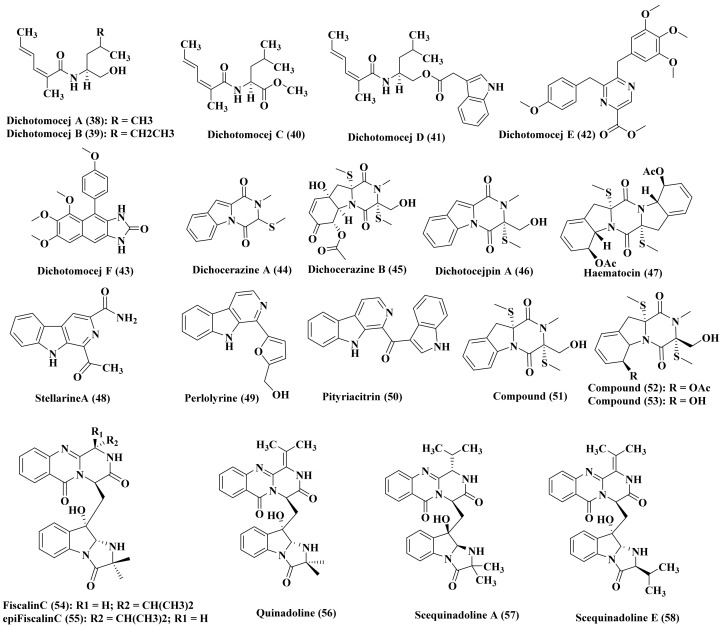
Alkaloids isolated from *Dichotomomyces* species.

*Dichotomomyces cejpii*, a marine-derived fungus, was isolated from the inner tissue of the soft coral *Lobophytum crassum* which was acquired from Hainan Sanya National Coral Reef Reserve, P. R. China. It represents a rich source of bioactive alkaloids particularly after the addition of l-phenylalanine and l-tryptophan to the culture broth comprising dichotomocejs A–F (38–43), dichocerazines A–B (44–45), dichotocejpin A (46), haematocin (47), stellarine A (48), fiscalin C (49), *epi*-fiscalin C (50), perlolyrine (51), pityriacitrin (52), didehydrobisdethiobis (methylthio) gliotoxin (53), 6-acetylbis (methylthio) gliotoxin (54), and bisdethiobis (methylthio) gliotoxin (55) (Figure 4). While investigating the cytotoxic activity of the previous compounds versus RD (human rhabdomyosarcoma cell line) as well as HCT116 (human colon cancer cell line) dichotomocej A (38) showed a notable activity versus RD displaying IC_50_ equals to 39.1 µM however pityriacitrin (52) revealed effectiveness versus HCT116 with 35.1 μM as IC_50_. Additionally their antibacterial activity was evaluated versus *Staphylococcus aureus*, *Pseudomonas aeruginosa,* as well as *Escherichia coli* and Bauman’s *acinetobacter* but unfortunately none of the tested compounds revealed any activity [24]. Besides, quinadoline A (56), scequinadolines A and E (57–58) were also isolated from *Dichotomomyces cejpii* and were assessed for their antiviral activity against dengue virus serotype 2 adopting the standard plaque assay. Only scequinadoline A (57) showed a considerable inhibitory potential versus the viral production (less than 50% at 50 μM) with values close to that of andrographlide, the positive control [25].

### 2.9. Eurotium

**Figure 5:**
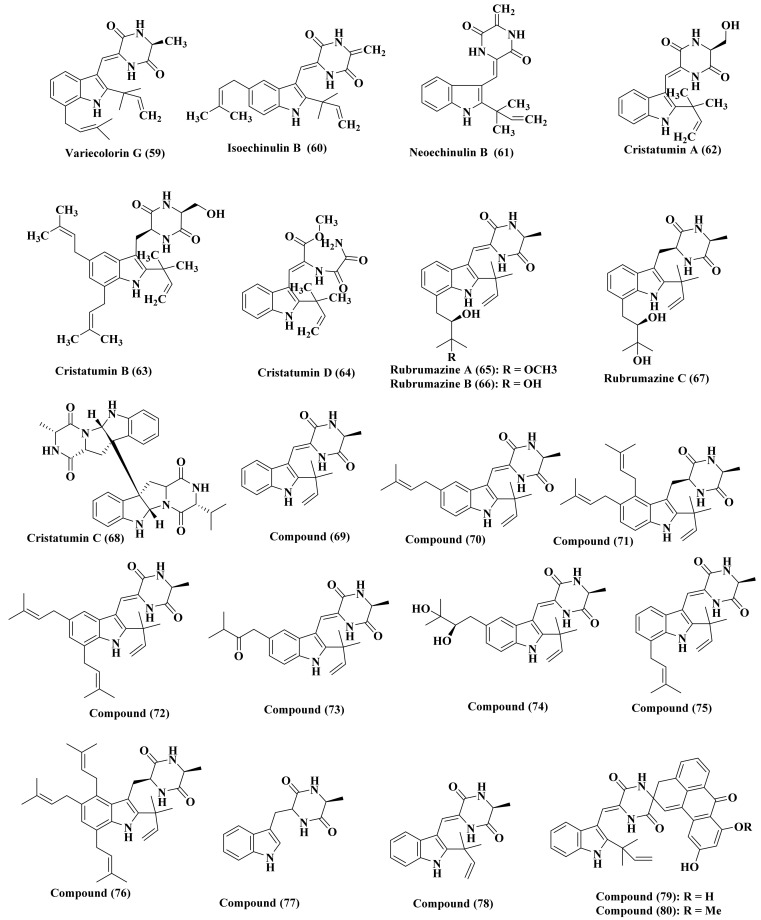
Alkaloids isolated from *Eurotium* species.

*Eurotium* species are considered to be a rich source of bioactive alkaloids where in depth phytochemical investigation of *Eurotium amstelodami*, marine -derived rhizospheric soil, yielded the isolation of new alkaloids possessing diketopiperazine indole skeleton which are variecolorin G (59), isoechinulin B (60) in addition to neoechinulin B (61) [26]. Besides, cristatumins A–D (62–65), four new alkaloids with indole moiety, are isolated from *Eurotium cristatum.* These compounds were assessed for their antibacterial and cytotoxic activity. Cristatumin A (62) exerted a considerable antimicrobial potential against *Escherichia coli;* however, cristatumin B (63) displayed a reasonable cytotoxic activity versus brine shrimp [27]. Additionally, a series of alkaloids were isolated from *Eurotium rubrum*, some of which are new and others were previously isolated. These alkaloids are represented by isoechinulin class with indolediketopiperazine skeleton which are rubrumazines A–C (66–68) that are newly isolated in addition to compounds (69–80). These compounds showed cytotoxicity employing brine shrimp lethal test with antibacterial activity showing different levels (Figure 5) [28].

**Figure 6:**
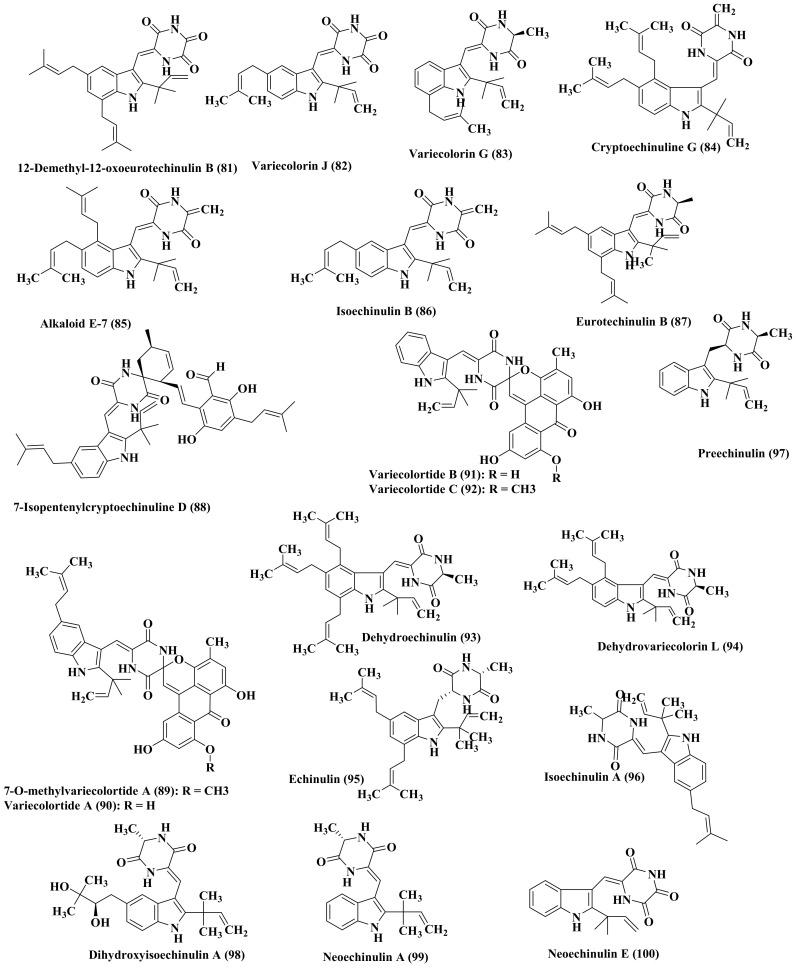
Alkaloids isolated from *Eurotium* species (Cont’d).

Additionally, more alkaloids were isolated from *Hibiscus tiliaceus*-derived *Eurotium rubrum* which are variecolorin G (59), 12-demethyl-12-oxoeurotechinulin B (81), variecolorin J (82), variecolorin H (83), cryptoechinuline G (84), alkaloid E-7 (85), isoechinulin B (86) eurotechinulin B (87), 7-isopentenylcryptoechinuline D (88). These compounds showed no antibacterial activity versus *Escherichia coli* and *Staphylococcus aureus* and weak antifungal activity against the examined fungi which are *Alternaria brassicae*, *Fusarium oxysporium,* and *Physalospora piricola*. The compounds were evaluated for their cytotoxic activity versus a number of cell lines namely, Hela, Du145, SMMC7721, MCF-7, SW1990, NCI-H460. 12-Demethyl-12-oxoeurotechinulin B (81), variecolorin G (59), and alkaloid E-7 (85) displayed a potent activity versus one or two of these cell lines with IC_50_ ranging between 20 and 30 μg/mL [29]. Besides, 7-*O*-methylvariecolortide A (89), a new alkaloid possessing spirocyclic diketopiperazine structure, in addition to variecolortides A–C (90–92) was isolated from the liquid fermentation cultures of *E. rubrum* [30]. Dehydroechinulin (93) and dehydrovariecolorin L (94), two new alkaloids of dioxopiperazine skeleton, were additionally isolated from *E. rubrum* together with echinuline (95), isoechinulin A (96), preechinulin (97), dihydroxyisoechinulin A (98), neoechinulin A (99), and E (100) in addition to cryptoechinuline D (101). Compounds (100–101) exhibited a potent antioxidant activity with IC_50_ values equal to 46.0 and 23.6 μM, respectively that are superior to butylated hydroxy-toluene (IC_50_ = 82.6 μM). Either of the two compounds (93–94) showed cytotoxic activity versus A-549, P-388 as well as HL-60 cell lines (Figure 6) [31].

**Figure 7:**
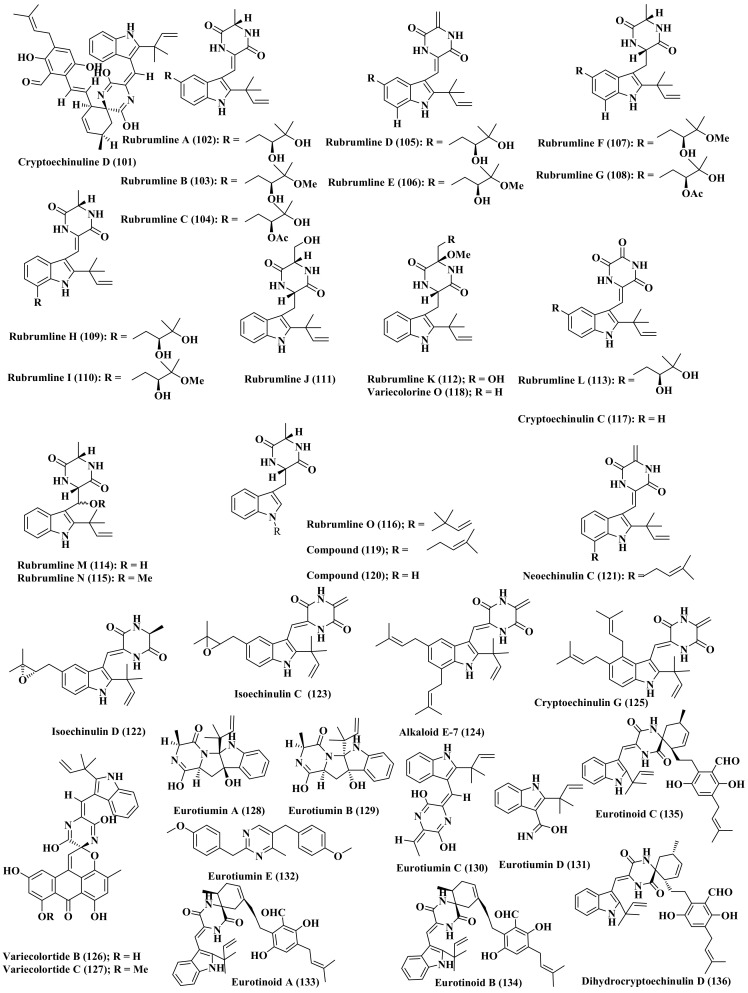
Alkaloids isolated from *Eurotium* species.

Additionally, a group of 15 new prenylated indole diketopiperazine, namely rubrumlines A-O (102–116) was isolated from *E. rubrum* (Figure 7) in addition to cryptoechinulin C (117), variecolorine O (118), echinuline (95), 3-methyl-6-[[1-(3-methyl-2-butenyl)-1H-indol-3-yl]methyl]- 2,5-piperazinedione (119), cyclo-alanyl-tryptophyl indole (120), and neoechinulin C (121) [10]. Rubrumline D (105), variecolorine O (118), neoechinulin C (121), isoechinulin A (96) and neoechinulin B (61) exerted a promising antiviral prohibition against influenza A/WSN/33 virus with IC_50_ of 126, 68.8, 51.2, 42.7, and 27.4 μg/mL, respectively. Neoechinulin B (61) showed a potent antiviral suppression in MDCK cells infected with H1N1 virus [10].

Additionally, changing the culture medium upon which the *E. rubrum*, isolated from a South China Sea sediment sample, grows greatly affected the produced secondary metabolites with subsequent influence on its bioactivity. Fungal extract obtained upon cultivating the fungus on wheat media showed more potent melanin synthesis inhibitory potential compared to that obtained while culturing the fungus on Czapek-Dox agar medium. A new diketopiperazine, isoechinulin D (122), and isoechinulin C (123), alkaloid E-7 (124), cryptoechinulin G (125) (Figure 7) in addition to echinuline (95), isoechinulin B (60), rubrumiline A (102), rubrumiline D (105), neoechinulin A (99) were isolated from the fungal extract cultured on a wheat medium [32]. Upon testing their melanin synthesis inhibitory effects, most of them showed melanogenesis inhibition using B16 melanoma cells with IC_50_ values ranging between 2.4 and 80 µM except for rubrumiline A (102) and neoechinulin A (99) which are inactive. On the contrary alkaloid E-7 exhibited the highest activity with IC_50_ value equal to 2.4 µM. This could be attributed to the presence of the prenyl groups at C-2, C-5, and C-7, the vinyl group at C-12 to C-25 and the sp2 carbons at C-8 and C9 that showed great importance to the activity based upon structure activity relationship study [32].

An additional study was performed on one of *Eurotium* sp. to assess the antifouling activity of its isolated indole alkaloids, neoechinulin A (99) and echinuline (95). The two compounds showed a notable inhibition to barnacle larval settlement with EC_50_ values equal to 15.0 and 17.5 μg/mL, respectively. Meanwhile, dihydroxyisoechinulin A (98) displayed a weak antifouling activity, however, variecolortide B (126), variecolortide C (127), and 7-O-methylvariecolortide A (89) isolated from the species were inactive [33].

Five new alkaloids namely, eurotiumins A–E (128–132) were isolated from the marine-associated *Eurotium* sp. Eurotiumins A–C (128–130) are 2,5-diketopiperazine alkaloids possessing a prenylated indole moiety meanwhile eurotiumin E is a new bis-benzyl pyrimidine derivative. Eurotiumins A–B (128–129) showed moderate antioxidant potential as evidenced by their free radical scavenging activity versus DPPH displaying IC_50_ values of 37 and 69 μM, respectively meanwhile eurotiumins C (130) and E (132) are highly potent with IC_50_ values of 13 and 19 μM, respectively. However, eurotiumin D (131) is ineffective with IC_50_ > 100 μM. Additionally, variecolorin G (59), isoechinulin A (96), variecolorin O (118), neoechinulin B (61), echinuline (95) are also effective antioxidant agents with IC_50_ of 4, 3, 24, 13, and 18 µM, respectively superior to that of ascorbic acid with IC_50_ equals 25 µM. All the new eurotiumins showed no anticancer effect versus SF-268 and HepG2 cell lines using SRB method in vitro [34].

Besides, eurotinoids A–C (133–135), three pairs of spirocyclic alkaloids enantiomers in addition to racemate dihydrocryptoechinulin D (136) were also isolated from *Eurotium* sp. All the compounds revealed potent radical scavenging potential versus DPPH showing IC_50_ values between 3.7 and 24.9 µM that are more effective than ascorbic acid. Besides, compound (+)-dihydrocryptoechinulin D revealed a moderate cytotoxic effect versus SF-268 and HepG2 cell lines with IC_50_ values of 51.7 and 49.9 µM, respectively meanwhile (–)-dihydrocryptoechinulin D showed IC_50_ of 97.3 and 98.7 µM, respectively [35].

### 2.10. Eutypella

**Figure 8:**
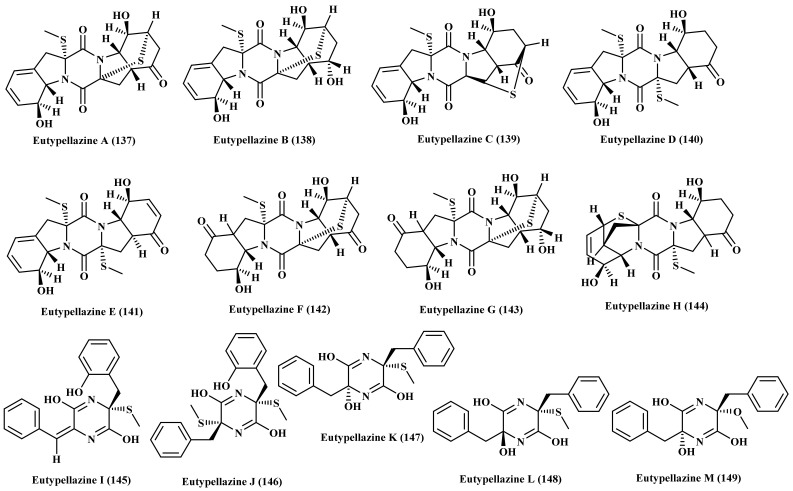
Alkaloids isolated from *Eutypella* species.

In depth phytochemical investigation of deep-sea-derived fungus *Eutypella*, *d*eep sea marine sediment, collected with TV-multicore from South Atlantic Ocean, resulted in the isolation and structural elucidation of thirteen new thiodiketopiperazine-type alkaloids, eutypellazines A–M (137–149) (Figure 8). Their structures were further confirmed via Mosher’s reaction, ECD data, and X-ray single-crystal diffraction for actual determination of the absolute configuration. The isolated compounds were assessed for their anti-HIV potential (human immunodeficiency virus type (1) using pNL4.3.Env-.Luc co-transfected 293T cells. Most of the new compounds revealed significant anti-HIV effect with IC_50_ ranging between 3.2 and 18.2 μM with eutypellazine E (141) revealing the highest potency (IC_50_ = 3.2 μM) [36].

### 2.11. Exophiala

**Figure 9:**
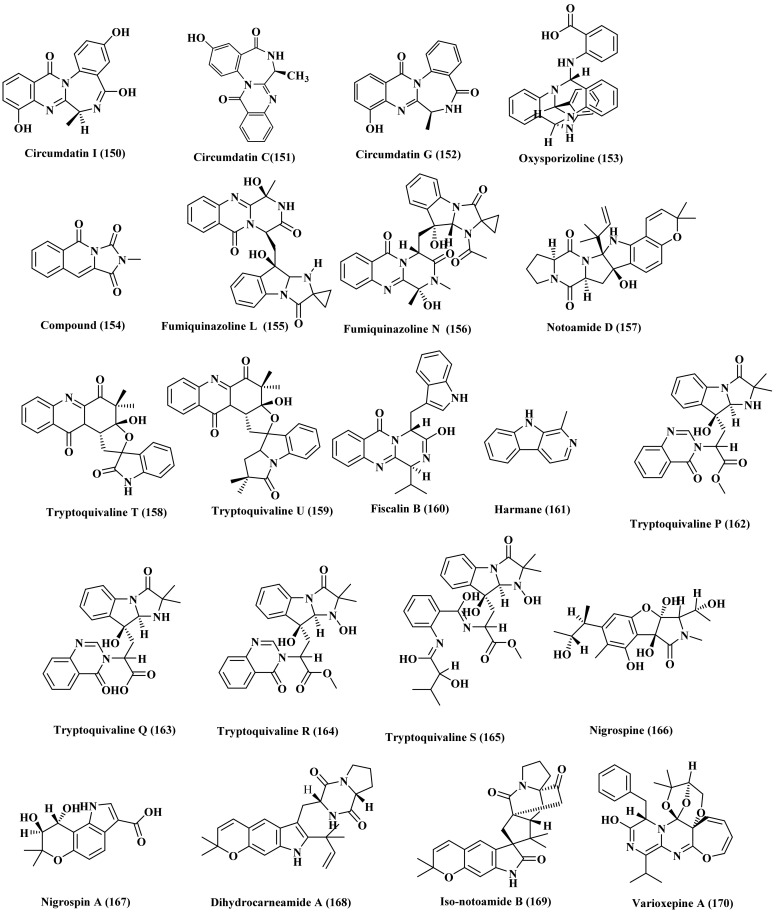
Alkaloids isolated from *Exophiala*, *Fusarium*, *Hypocrea*, *Microsphaeropsis*, *Microsporum*, *Microsporum*, *Nigrospora*, and *Paecilomyces* species.

A new benzodiazepine alkaloid namely circumdatin I (150) in addition to circumdatin C (151) and G (152) were isolated from the marine-associated fungus *Exophiala*. They were examined for their UV-A protective behavior where they all showed a potent activity with EC_50_ values equal to 98, 101, and 105 μM, respectively showing higher potency when compared to oxybenzone (ED_50_, 350 μM), which was used as a positive control being a commonly used sunscreen agent (Figure 9) [37].

### 2.12. Fusarium

Phytochemical investigation of the crude extract of marine-associated fungus, *Fusarium oxysporum*, isolated from the marine mudflat collected at Suncheon Bay, Korea, resulted in the isolation of a new polycyclic quinazoline alkaloid, oxysporizoline (153) that revealed an antibacterial activity against MRSA and MDRSA with MIC equal to 6.25 µg/mL in addition to notable antioxidant potential manifested by its observable radical scavenging effect versus DPPH with IC_50_ equals to 10 µM (Figure 9) [38].

### 2.13. Hypocrea

Fractionation and purification of the different fractions obtained from the extract obtained from marine-derived fungus *Hypocrea virens*, isolated from *R. apiculata* of Shatian country, Guangxi province, China, resulted in the isolation of a new alkaloid termed 2-methylimidazo [1,5-b]isoquinoline-1,3,5(2H)-trione (154) (Figure 9) [39].

### 2.14. Microsphaeropsis

Bioassay-guided fractionation of a marine sediment-derived fungus, *Microsphaeropsis*, which was collected from the Huanghua in the Bohai Sea, resulted in the isolation of three alkaloids which are fumiquinazolines L (155) and N (156) and notoamide D (157) (Figure 9) [40].

### 2.15. Microsporum

Neoechinulin A (99), a prenylated indole alkaloid, was isolated from the extract of the culture broth of marine-derived Microsporum sp., isolated from the surface of a marine red alga *Lomentaria catenata*, collected at Guryongpo, NamGu, PoHang in Republic of Korea. The alkaloid revealed a potent cytotoxic effect on HeLa cells inducing apoptosis manifested by the p21, p53, Bax, Bcl-2, caspase 3, and caspase 9 expressions. Neoechinulin A (99) effectively enhances cell apoptosis via the downregulation of Bcl-2 expression with concomitant upregulation of Bax expression and enhancement of caspase-3 as evidenced by the Western blot (Figure 9) [41].

### 2.16. Neosartorya

Phytochemical investigation of the ethyl acetate extract obtained from the fermentation broth of marine-derived fungus *Neosartorya fischeri*, isolated from marine mud in the intertidal zone of Hainan Province of China, resulted in the characterization of three alkaloids, two of which are new namely, tryptoquivaline T (158), tryptoquivaline U (159) in addition to fiscalin B (160). All the tested compounds showed notable cytotoxic potential evidenced by induction of HL-60 cells apoptosis displaying IC_50_ values of 82.3, 90.0, and 8.88 μM respectively [42]. Furthermore, harmane (161) was isolated from the sponge derived fungus *Neosartorya tsunodae*, isolated from the marine sponges *Aka coralliphaga*, collected at the coral reef of Similan Islands, Phang Nga Provice [43]. Besides, four new alkaloids were isolated from a marine-associated fungus, *Neosartorya* sp. which are tryptoquivalines P–S (162–165) (Figure 9) [44].

### 2.17. Nigrospora 

Two new alkaloids namely nigrospine (166) and nigrospin A (167) were isolated from *Nigrospora oryzae*, a marine-derived fungus isolated from a marine gorgonian *Verrucella umbraculum* collected in the South China Sea near Sanya City. The former is a pyrrolidinone alkaloid meanwhile the latter is indole type alkaloid. The absolute configuration of both compounds were determined employing Mosher reaction (Figure 9) [45].

### 2.18. Paecilomyces 

The marine-derived endophytic fungus *Paecilomyces variotii*, isolated from *Grateloupia turuturu*, a marine red alga collected from the coast of Qingdao, China, was comprehensively investigated using various chromatographic and spectral techniques and led to the isolation of dihydrocarneamide A (168), iso-notoamide B (169) which were two new prenylated indole alkaloids in addition to varioxepine A (170), a new 3H-oxepine-containing alkaloid. Dihydrocarneamide A (168) and iso-notoamide B (169) displayed weak cytotoxic potential versus NCI-H460 cell lines (human large cell lung carcinoma) with IC_50_ values equal to 69.3 and 55.9 μmol/L, respectively [46] meanwhile varioxepine A (170) showed a potent antifungal activity versus *Fusarium graminearum* (Figure 9) [47].

### 2.19. Penicillium

**Figure 10:**
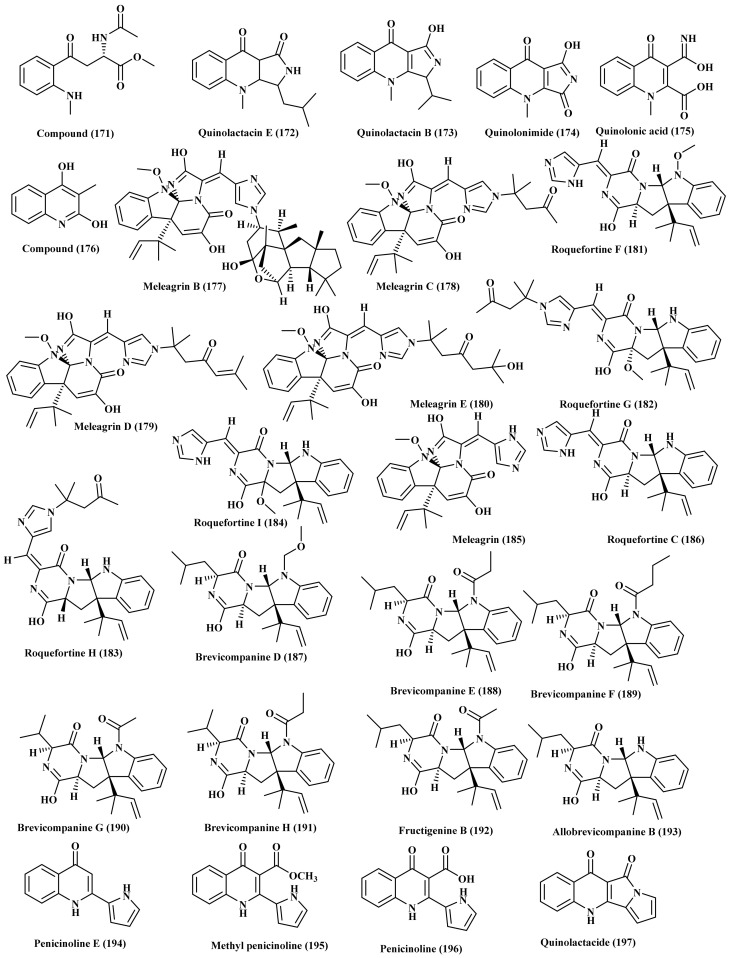
Alkaloids isolated from *Penicillium* species.

Two new alkaloids named (S)-methyl 2-acetamido-4-(2-(methylamino) phenyl)-4-oxobutanoate (171) and quinolactacin E (172) in addition to four known compounds quinolactacin B (173) quinolonimide (174) quinolonic acid (175) and 4-hydroxy-3-methyl-2(1H)-quinolinone (176) were isolated from the marine sponge-associated *Penicillium* species, obtained from a *Callyspongia* sp. sponge, which was collected from the sea area near Xuwen County, Guangdong Province, China. All the compounds except quinolactacin E (172) were evaluated for their cytotoxicity versus six human cancer cells as well as their antibacterial behavior against five pathogenic bacteria. None of the tested compounds showed inhibitory potential when examined at concentrations of 5 µM in the preliminary screening [48]. In addition eight new alkaloids comprising meleagrin B–E (177–180), roquefortine F–I (181–184), and two previously isolated compounds meleagrin (185) as well as roquefortine C (186) were isolated from *Penicillium* species isolated from deep ocean marine sediment. The compounds were assessed for their cytotoxic activity versus four cell lines HL-60, A-549, BEL-7402, and MOLT-4. Only meleagrin B (177) displayed moderate cytotoxic activity with IC_50_ ranging between 6.7, 2.7, 1.8, and 2.9 μM inducing HL-60 cell apoptosis meanwhile meleagrin (185) arrested the cell cycle through G2/M phase that could be interpreted by virtue of the different substitutions on the imidazole ring [49]. Furthermore, brevicompanines D–H (187–191), new alkaloids of diketopiperazine type, and two known alkaloids fructigenine B (192) and allobrevicompanine B (193), were also isolated from *Penicillium* species obtained from deep ocean sediment (Figure 10). Brevicompanines E (188) and H (191) effectively prohibited H lipopolysaccharide (LPS)-stimulated nitric oxide formation in BV2 microglial cells displaying IC_50_ values of 27 and 45 μg/mL, respectively [50]. Furthermore, penicinoline E (194), a new quinolinone alkaloid with a pyrole ring, and three previously reported alkaloids, methyl penicinoline (195), penicinoline (196), and quinolactacide (197) (Figure 10) were also obtained from a marine-associated *Penicillium*, isolated from a marine sediments in Jiaozhou bay in China. Only methyl penicinoline (195) and penicinoline (196) displayed notable cytotoxicity versus Hep G2 cells with IC_50_ 11.2 and 13.2 μM, respectively [51].

**Figure 11:**
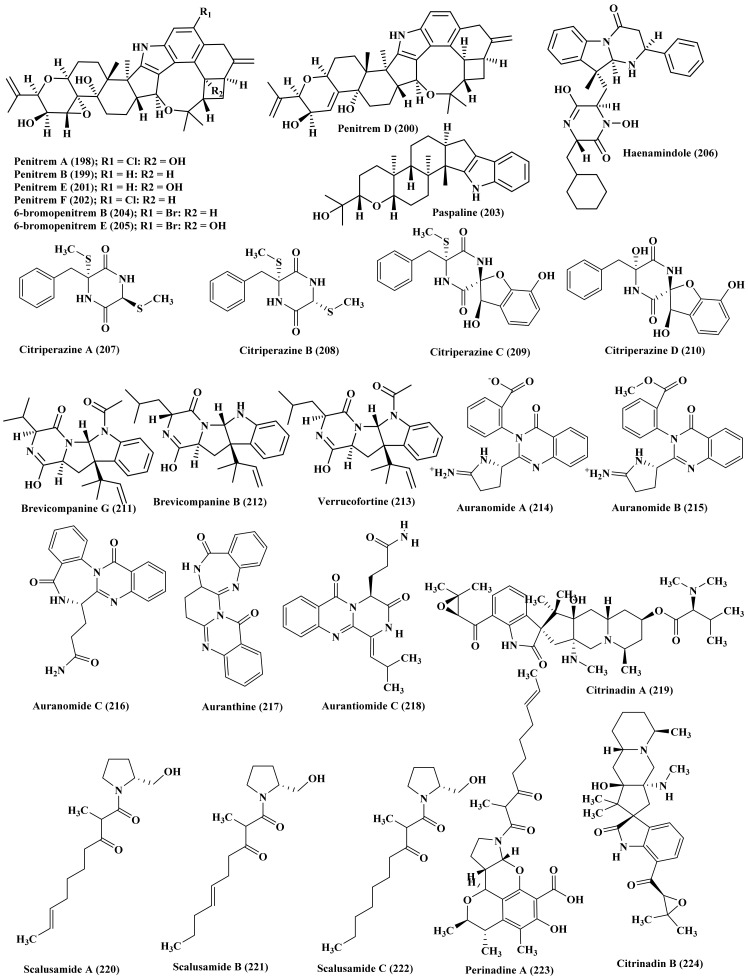
Alkaloids isolated from *P. aurantiogriseum*, *P. chrysogenum*, *P. citrinum* and other miscellaneous *Penicillium* species.

Additionally, marine-derived *Penicillium*, isolated from a marine sediment collected from the coast of Haenam, Korea, is highly popular by many bioactive metabolites evidenced by the isolation of penitrems A, B, D, E, and F (198–202), indole diterpene alkaloids, as well as paspaline (203) from its fermentation medium. Meanwhile the addition of potassium bromide to the fermentation medium resulted in the additional isolation of 6-bromopenitrem B (204), new alkaloid, and a known one that is 6-bromopenitrem E (205) (Figure 11). All the isolated compounds displayed notable anti-migratory, anti-invasive, and anti-proliferative potential against human breast cancer cells MCF-7 cells exhibiting IC_50_ ranging between 5.5 and 19.3 μM. Besides, penitrem B (199) revealed an effective cytotoxic behavior versus NCI-60 DTP human. Furthermore, the nematode *Caenorhabditis elegans* was used to assess the brain’s Maxi-K (BK) channel inhibitory activity and toxicity in vivo. Penitrem A (198) and 6-Bromopenitrem E (205) revealed a BK channel inhibition, comparable to that of a knockout strain. They showed the highest potency as a tremorgen reversing the pattern in a manner equivalent to the knockout strain [52]. Moreover, haenamindole (206), diketopiperazine with unusual structure possessing both benzyl-hydroxypiperazindione and phenyl-pyrimidoindole moieties, was isolated from the marine-associated *Penicillium* fungus. The structure and the absolute configuration were comprehensively confirmed based upon NMR, MS in addition to Marfey’s reaction [53]. Four new alkaloids, citriperazines A–D (207–210) of diketopiperazine class were also isolated from algae-derived *Penicillium* fungus, isolated from Vietnamese marine brown algae *Padina* sp., in which the structures were determined using different spectroscopic techniques meanwhile the absolute configuration was determined based upon ECD calculations. None of the compounds showed activity when assessed for their cytotoxic potential versus human prostate cancer cells [54].

Additionally, brevicompanine G (211), diketopiperazine alkaloid was isolated from the ethyl extract of a coral-derived *Penicillium* fungus, isolated from a piece of the inner tissues of a fresh soft coral of the genus *Alcyonium* which was collected from the Sanya Bay, Hainan Island, China. The isolated compound was evaluated for its iso-citrate dehydrogenase inhibitory potential but it showed no activity at 20 µM. Besides, the compound was examined for its cytotoxic effect versus a number of cancer cells namely, SW-480 (colon cancer), A-549 (lung cancer), HL-60 (acute leukemia), HEP3B (hepatic cancer), MM231 (breast cancer), and NCM460 (normal colonic epithelial cell) but it revealed no activity [55]. Brevicompanine B (212), and verrucofortine (213), two new prenylated indole alkaloids, were isolated from *Penicillium* marine-derived fungus. The isolated compounds showed no cytotoxic effect versus mouse Hepa lclc7 cells at 20 nM [56]. Auranomides A–C (214–216), three new alkaloids, in addition to auranthine (218) and aurantiomide C (218), two known compounds, were isolated from a marine-associated *P. aurantiogriseum*, isolated from marine mud of the Bohai Sea. Auranomides A–C (214–216) displayed notable cytotoxicity versus human tumor cells where auranomide B (215) revealed the highest potency with IC_50_ equals to 0.097 µmol/mL versus HEPG2 cells [57]. Two alkaloids, terremide D, a new one, and methyl 3,4,5-trimethoxy-2-(2-(nicotinamido) benzamido) benzoate, a known alkaloid were isolated from deep-sea-derived fungus *P. chrysogenum* in addition to other miscellaneous compounds (Figure 11) [58]. Citrinadin A (219), a novel pentacyclic alkaloid, scalusamides A–C (220–222), three new pyrrolidine alkaloids, perinadine A (223), a novel tetracyclic alkaloid, as well as citrinadin B (224) were isolated from marine-derived fungus *P. citrinum* (Figure 11). Scalusamides A–C (220–222) exhibited significant antimicrobial activity [59,60,61,62].

**Figure 12:**
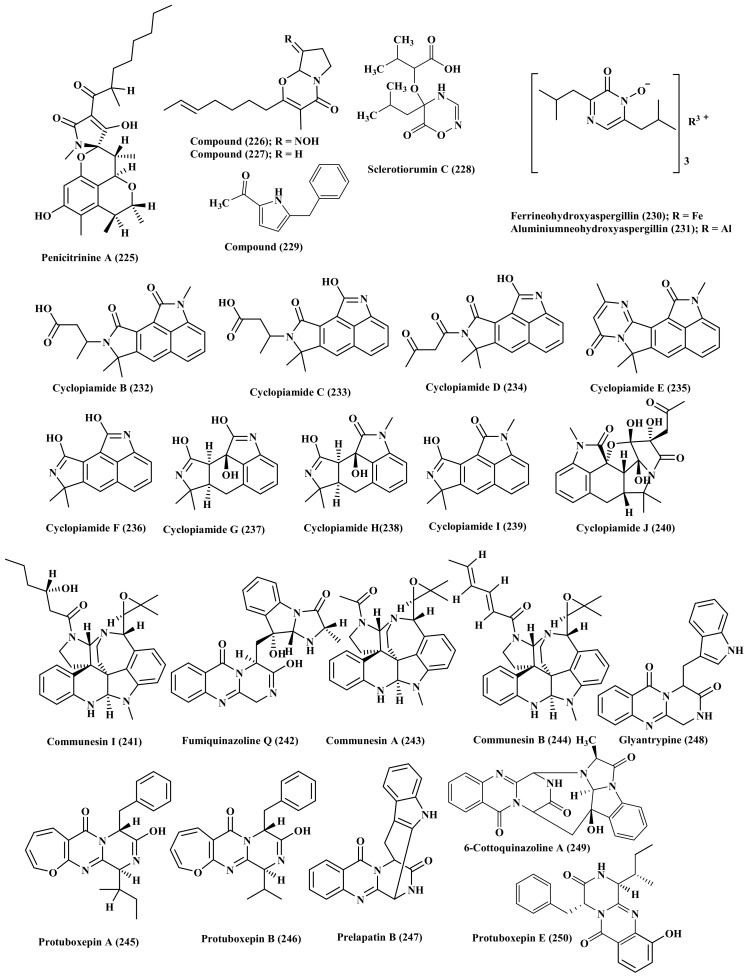
Alkaloids isolated from *P. citrinum*, *P. commune,* and *P. expansum.*

Additionally, penicitrinine A (225), a novel compound possessing spiro skeleton was also isolated from *P. citrinum*, isolated from marine sediments collected from Langqi Island, Fujian, China. It revealed a potent anti-proliferative activity in A-375 (human malignant melanoma) cells where it significantly enhanced the cell apoptosis via reducing Bcl-2 expression and enhancing Bax expression. Besides, it reduced the metastatic potential of A-375 cells by virtue of controlling MMP-9 and TIMP-1 expression [63]. The activation of *P. citrinum* silent genes to afford different bioactive secondary metabolites was induced via the addition of 50 μM of scandium chloride to the fermentation medium. Consequently, pyrrolidine alkaloids, (E)-2-(hept-5 -enyl)-3-methyl-4-oxo-6,7,8,8a- tetrahydro -4H-pyrrolo [2,1-b]-1,3-oxazine (226), and (E) -2-(-hept-5-enyl)-8-(hydroxyimino)- 3-methyl-4-oxo-6,7,8,8a-tetrahydro-4H-pyrrolo [2,1-b]-1,3-oxazine (227) were isolated which were not detected without the addition of ScCl_3_. These compounds showed no cytotoxicity versus SKMEL-2 (human skin cancer), HepG2 (human liver cancer), XF-498 (human CNS cancer), HCT115 (human colon cancer), and MCF-7 (human breast cancer) [64]. Noteworthy to highlight that the co-culture of *P. citrinum* with *Aspergillus sclerotiorum* resulted in the isolation of sclerotiorumin C (228), a novel oxadiazin derivative, in addition to 1-(4-benzyl-1H-pyrrol-3-yl)ethanone (229), ferrineohydroxyaspergillin (230), and aluminiumneohydroxyaspergillin (231). The latter showed a selective cytotoxic potential versus U937 cell line (human histiocytic lymphoma) with IC_50_ equals 4.2 µM and considerable toxicity versus brine shrimp with LC_50_ equals 6.1 µM. On the contrary it enhanced the growth of *Staphylococcus aureus* and its biofilm formation as well (Figure 12) [65].

*P. commune* associated with deep sea obtained sediments collected from the South China Sea, Sansha City is a rich source of oxindole alkaloids where nine new compounds, cyclopiamides B–J (232–240) were isolated and structurally elucidated using different spectroscopic techniques in addition their absolute configurations were determined using single crystal X-ray diffraction and ECD techniques [66]. *P. expansum* was derived from a marine source from which new alkaloids were isolated, communesin I (241) and fumiquinazoline Q (242) in addition to known compounds which are communesin A–B (243–244), protuboxepin A–B (245–246), prelapatin B (247), glyantrypine (248), 6cottoquinazoline A (249), and protuboxepin E (250). Most of the isolated compounds displayed a potent activity on the bradycardia induced by astemizole (ASM) in the heart rate in live zebra fish model in addition to exerting a potent vasculogenetic activity in vasculogenesis (Figure 12) [67]. In addition, *P. granulatum* is a good source of alkaloids from which roquefortine J (251), a novel compound, was isolated in addition to 16-hydroxyroquefortine C (252), roquefortine C (186), roquefortine F (181), and meleagrin (185). Only meleagrin (185) revealed a notable anti-proliferative potential versus HepG2 tumor cells showing IC_50_ value of 7.0 µM [68].

**Figure 13:**
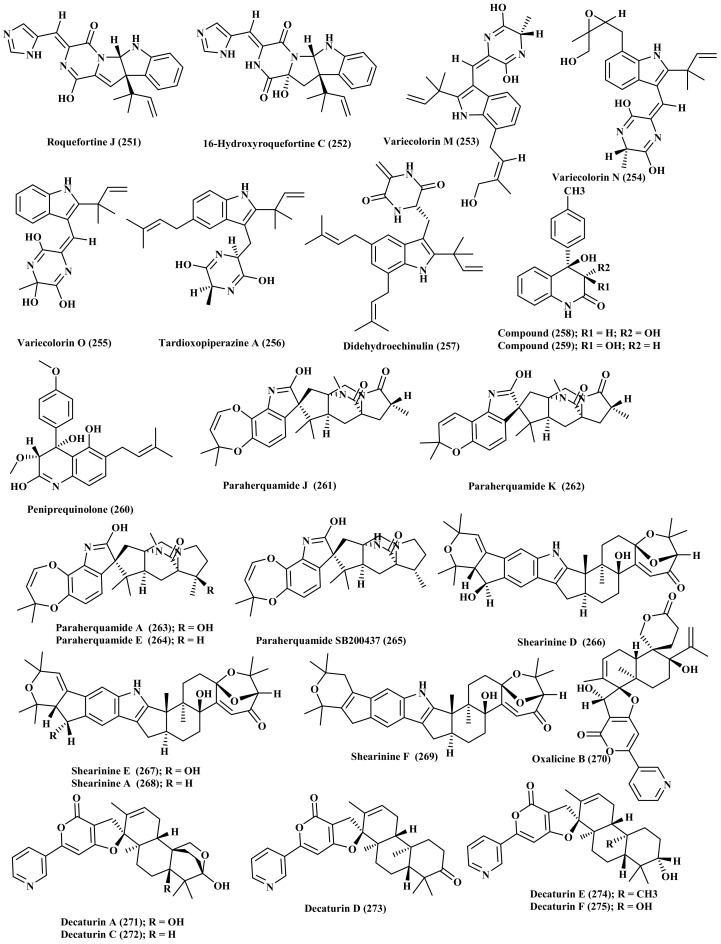
Alkaloids isolated from *P. granulatum*, *P. griseofulvum*, *P. janczewskii*, *P. janthinellum*, and *P. oxalicum*.

*P. griseofulvum* derived from the sediment of a deep ocean represents a rich source of bioactive indole alkaloids from which three new alkaloids, variecolorins M–O (253–255), and other known ones were isolated. These known alkaloids are tardioxopiperazine A (256), didehydroechinulin (257), neoechinulin A (99), isoechinulin B (60), neoechinulin (61), variecolorin H (71), echinuline (95), preechinulin (97). Variecolorins M–O (253–255) revealed a weak antioxidant potential manifested by their IC_50_ values in DPPH radical scavenging capacity assay which are 135, 120, and 91 µM, respectively meanwhile none of them exhibited cytotoxic effect versus BEL-7402, HL-60, and A-549 cell lines as examined using both MTT and SRB assays (Figure 13) [69].

New diastereomeric quinolinone alkaloids, 3S*,4R*-dihydroxy-4-(4′methoxyphenyl)-3,4-dihydro-2(1H)-quinolinone (258) and 3R*,4R*dihydroxy-4-(4′-methoxyphenyl)-3,4-dihydro-2(1H)-quinolinone (259) were isolated from *P. janczewskii* derived from marine sample along with two previously isolated alkaloids, peniprequinolone (260) and 3-methoxy-4-hydroxy-4-(4′methoxyphenyl)-3,4-dihydro-2 (1H)-quinolinone. Compound (259) only showed cytotoxic effect on SKOV-3 cells (Figure 13) [70].

Moreover, *P. janthinellum*, marine rhizosphere soil-derived fungus, acts as a rich source of prenylated indole alkaloids, two of which are new compounds, paraherquamide J–K (261–262) whereas paraherquamides A, E, and SB200437 (263–265) were previously isolated. Unfortunately none of the isolated compounds revealed any antibacterial, topoisomerase I inhibitory effects or lethal effects versus brine shrimp *Artemia salina* [71]. Besides, shearinines D–F (266–268), three new alkaloids possessing indole moiety, together with shearinine A (269), a known compound, were isolated from *P. janthinellum* marine-derived fungus. Shearinines A (269), D (266), and E (267) enhance apoptosis in HL-60 cells, meanwhile shearinine E (267) prohibits EGF-stimulated malignant change of JB6 P+ Cl 41 cells (Figure 13) [72]. In depth phytochemical investigations of the mycelia of *P. oxalicum* afforded oxalicine B (270), decaturin A (271), and decaturin C-F (272–275) in which decaturins E (274) and F (275) were isolated and identified as new compounds [73].

Three new indole alkaloids containing prenyl group, penipalines A (276) and B (277), *β-*carbolines in addition to penipaline C (278), indole carbaldehyde derivative, were isolated from *P. paneum* derived from deep-sea-sediment. Additional two known alkaloids, dimethyl-1H-*β*-carboline-3-carboxylic acid (279) and 1,7-dihydro-7,7-dimethylpy-rano[2,3-g] indole-3-carbaldehyde (280) were also isolated. Upon testing the cytotoxic effect of the new compounds versus A-549 and HCT-116 cell lines, only penipaline B (277) and C (278) showed notable effect versus both cell lines with IC_50_ of 20.4 and 21.5 μM, respectively against A-549 and 14.8 and 18.5 for them, respectively versus HCT-116 [74].

In addition, penipanoid A (281), a triazole carboxylic acid of novel structure, penipanoids B (282) and C (283), two new alkaloids possessing quinazolinone moiety together with a quinazolinone derivative (284) were also isolated from a marine sediment-associated *P. paneum.* Compounds (281) and (283) exerted certain antimicrobial and cytotoxic activity [75]. Regarding the marine-derived *P. purpurogenum* mutant, penicimutamides A–C (285–287), three carbamate-containing alkaloids and penicimutamides D–E (288–289), prenylated indole alkaloids and premalbrancheamide (290) were isolated. Their structures and absolute configuration were determined based upon spectroscopic data, X-ray crystallography, CD analyses, HPLC-MS, and HPLC-UV data [76,77].

**Figure 14:**
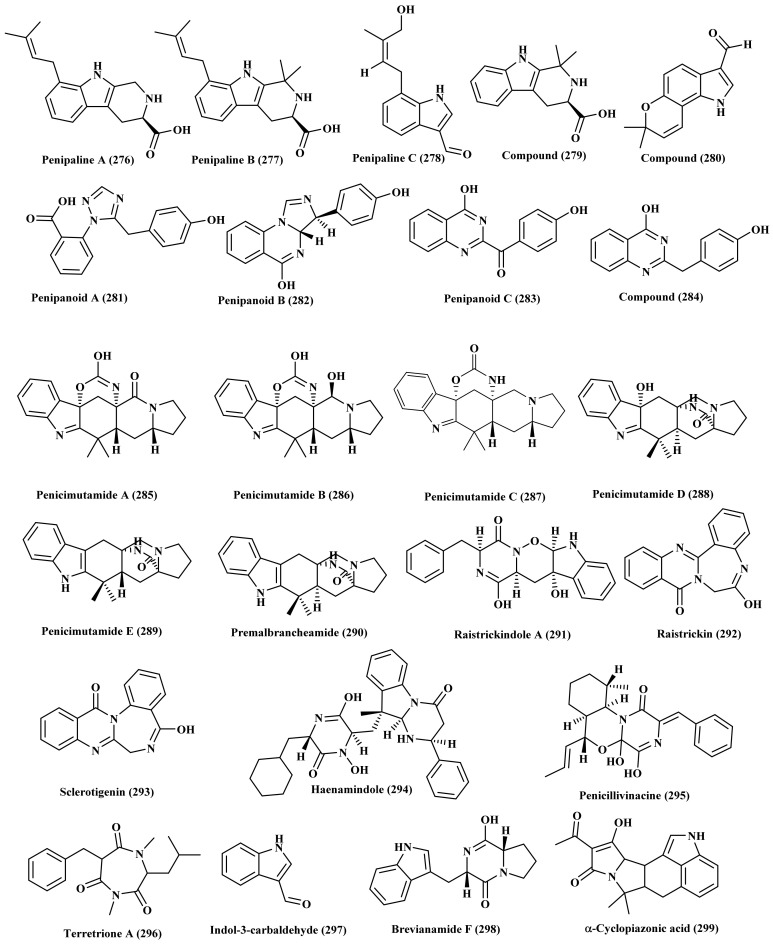
Alkaloids isolated from *P. paneum*, *P. purpurogenum*, *P. raistrickii*, and *P. vinaceum*.

In depth phytochemical investigation of *P. raistrickii*, a marine-derived fungus, led to the isolation of four alkaloids, two of which, raistrickindole A (291) and raistrickin (292) represent new alkaloids, the former possess indole diketopiperazine group meanwhile the latter contains benzodiazepine moiety. Additionally, two new alkaloids, sclerotigenin (293) and haenamindole (294) were isolated. Both compounds showed potent antiviral activity versus hepatitis C virus [78]. Furthermore, *P. vinaceum*, isolated from the marine sponge *Hyrtios erectus* collected from Yanb, contains a lot of alkaloids comprising a new one, penicillivinacine (295), in addition to other known compounds including terretrione A (296), indol-3-carbaldehyde (297), brevianamide F (298), α-cyclopiazonic acid (299) (Figure 14). Penicillivinacine (295) and terretrione A (296) showed a considerable anti-migratory potential versus the greatly metastatic MDA-MB-23 cells (human breast cancer cells) displaying IC_50_ values of 18.4 and 17.7 µM, respectively. In addition all the isolated compounds were tested for their antimicrobial activity against a panel of micro-organism including *Staphylococcus aureus*, *Escherichia coli,* and *Candida albicans*; Brevianamide F (298) showed antibacterial activity against *Staphylococcus aureus* displaying 19 mm as diameter of inhibition zone; meanwhile α-cyclopiazonic acid showed effect versus *E. coli* and with zone of inhibition equals to 20 mm. Both terretrione A (296) and brevianamide F (298) displayed inhibition zones of 27 and 25 mm against *Candida albicans*, respectively [79].

### 2.20. Pleosporales

**Figure 15:**
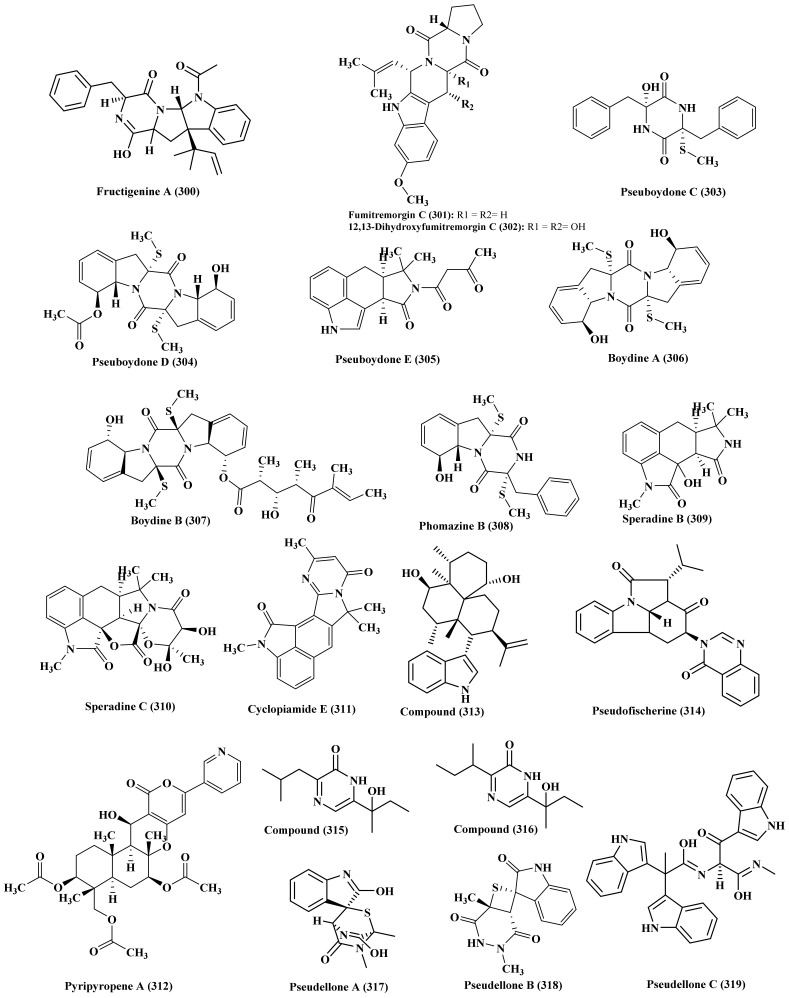
Alkaloids isolated from *Pleosporales* and *Pseudallescheria*.

A deep phytochemical exploration of the ethyl acetate extract obtained from a marine-derived fungus isolated from marine sediment collected from the Huanghua in the Bohai Sea, *Pleosporales* led to the isolation of three alkaloids of diketopiperazine type namely, fructigenine A (300), fructigenine B (192), and brevicompanine G (211). None of the isolated compounds revealed any antimicrobial activity when tested versus 16 pathogenic microbial strains (Figure 15) [80].

### 2.21. Pseudallescheria

Three dioxopiperazine alkaloids, 12R,13S-dihydroxyfumitremorgin (301), fumitremorgin C (302) and brevianamide F (298) were isolated from the marine-associated *Pseudallescheria* species. The three isolated compounds showed notable antimicrobial activity against *Staphylococcus aureus*, methicillin-resistant *S. aureus*, and multidrug-resistant *S. aureus* with minimum inhibitory concentration of 125 µg/mL for all compounds for all strains [81]. Furthermore, a soft-coral-derived *P. boydii*, isolated from the inner tissue of the soft coral *Lobophytum crassum* collected from Hainan Sanya National Coral Reef Reserve, P. R. China, is a rich source of alkaloids from which three new compounds were isolated namely, pseuboydones C, D (303–304) and pseuboydone E (305). In addition known compounds as boydine A (306), boydine B (307), haematocin (47), phomazine B (308), speradine B (309), speradine C (310), cyclopiamide E (311), pyripyropene A (312), 4-(1-hydroxy-1-methylpropyl)-2-isobutyl-pyrazin-2(1H)-one (313), pseudofischerine (314), 4-(1-hydroxy-1-methyl-propyl)-2-secbutylpyrazin-2(1H)-one (315), 24,25-dehydro-10,11-dihydro-20- hydroxyaflavinin (316) were isolated. Pseuboydone C (303), speradine C (310), 24,25-dehydro-10,11-dihydro-20-hydroxyaflavinin (316) showed cytotoxicity versus Sf9 cells with IC_50_ values 0.7, 0.9, and 0.5 μM respectively [82]. Meanwhile, pseudellones A–C (317–319) were isolated from a marine-associated fungus *P. ellipsoidea* that were determined based upon X-ray crystallography and ECD calculations (Figure 15) [83].

### 2.22. Scedosporium

**Figure 16:**
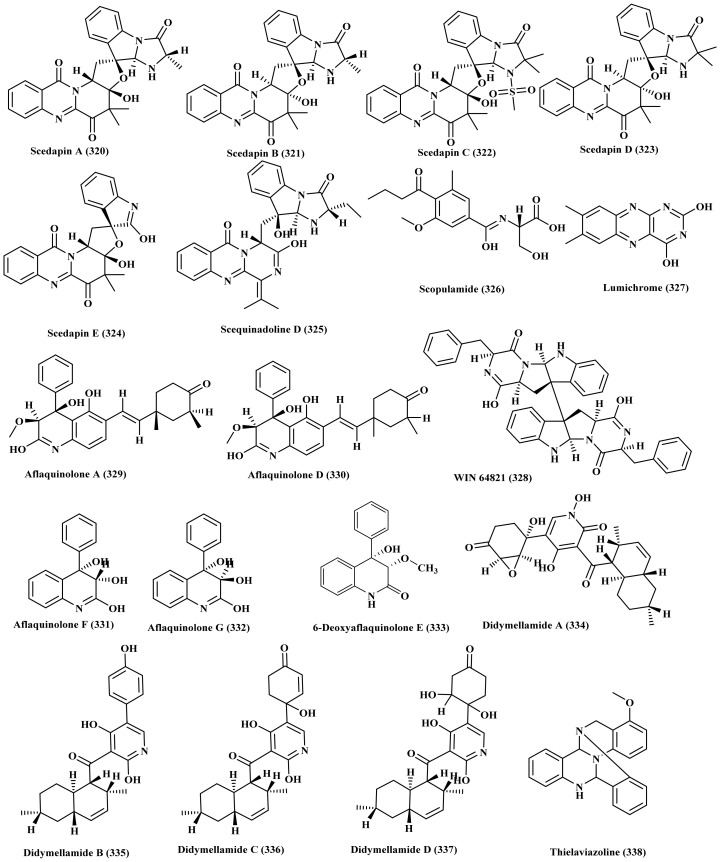
Alkaloids isolated from *Scedosporium*, *Scopulariopsis*, *Stagonosporopsis*, and *Thielavia*.

In depth phytochemical screening of the ethyl acetate extract of the marine fungus *S. apiospermum* fed by different amino acids led to the isolation of various new alkaloids comprising scedapins A–E (320–324) in addition to scequinadoline D (325). Both scedapin C (322) and scequinadoline D (325) showed promising antiviral potential versus hepatitis C (Figure 16) [84].

### 2.23. Scopulariopsis

In depth phytochemical investigation of *Scopulariopsis*, isolated from the fresh crushed inner tissues of the Red Sea hard coral *Stylophora* sp., led to the isolation of three alkaloids, one of which is new, scopulamide (326) in addition to two known alkaloids, lumichrome (327) and WIN 64,821 (328). They showed weak cytotoxic effect versus e L5178Y mouse lymphoma cell line [85]. In addition, six dihydroquinolin-2-one containing alkaloids namely, aflaquinolone A, D, F, G (329–332), and 6-deoxyaflaquinolone E (333) were also isolated from *Scopulariopsis.* All the isolated alkaloids showed antifouling effect against larval settlement of barnacle *Balanus amphitrite* additionally aflaquinolone A (329) showed a significant activity with EC_50_ value of 17.5 pM (Figure 16) [86].

### 2.24. Stagonosporopsis

New pyridone alkaloids namely, didymellamides A–D (334–337) were isolated from *Stagonosporopsis cucurbitacearum*, a marine-associated fungus isolated from the surface of an unidentified sponge collected off the coast of Atami-shi, Shizuoka Prefecture, Japan. Didymellamide A (334) inhibited the growth of azole-resistant and -sensitive *C. albicans*, *C. glabrata*, and *Cryptococcus neoformans* at concentrations of 1.6 or 3.1 μg/mL; meanwhile didymellamide B (335) inhibited only *C. neoformans* with an MIC of 6.3 μg/mL (Figure 16) [87].

### 2.25. Thielavia

Thielaviazoline (338) isolated from *Thielavia*, a marine-derived fungus isolated from the marine mudflat collected at Gomso Bay, Korea, displayed antimicrobial effect against MRSA (methicillin-resistant *Staphylococcus aureus*) and MDRSA (multidrug resistant *Staphylococcus aureus*) with MIC values equal to 6.25 and 12.5 µg/mL, respectively. In addition, it showed an effective radical-scavenging activity against 2,2-dipheny1–1-picrylhydrazyl (DPPH) displaying 11 µM as an IC_50_ (Figure 16) [88].

### 2.26. Westerdykella

**Figure 17:**
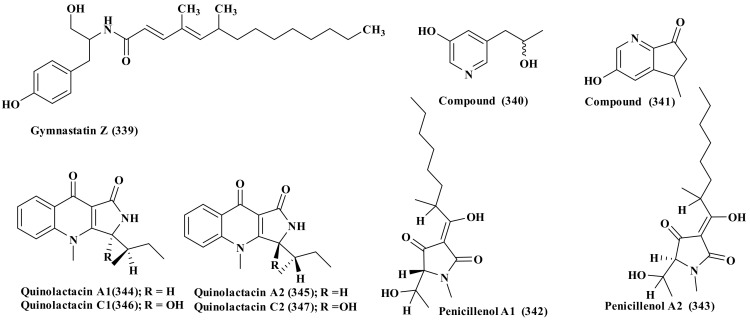
Alkaloids isolated from *Westerdykella* and *Xylariaceae.*

Gymnastatin Z (339), a new tyrosine-derived alkaloid was isolated from *Westerdykella dispersa*, obtained from marine sediments, which were collected at South China Sea, Guangzhou, Guangdong province, China. It showed substantial effect against *B. subtilis* with MIC equals 12.5 µg/mL. In addition, it showed certain inhibitory potential versus MCF-7, HepG2, A549, HT-29, and SGC-7901 cell lines with IC_50_ values ranging between 25.6 and 83.7 µM. (Figure 17) [89].

### 2.27. Xylariaceae

A marine-derived *Xylariaceae* species, isolated from the South China Sea gorgonian coral *Melitodes squamata*, is a rich source of alkaloids, from which 5-(2′-hydroxypropyl) yridine-3-ol (340), a new alkaloid in addition to seven known compounds termed 3-hydroxy-5-methyl-5,6-dihydro7H-cyclopenta[b]yridine-7-one (341), penicillenol A1 and A2 (342–343), quinolactacin A1, A2, C1, and C2 (344–347) was isolated (Figure 17). Compound (341) showed weak antimicrobial activity against *Bacillus subtilis* with an inhibitory zone of 8 mm, while the other compounds did not show obvious activity toward *B. subtilis* and *Escherichia coli*. Meanwhile compounds (344–346) revealed strong antifouling potential versus *Bugula neritina* larval settlement with an EC_50_ value of 6.21 μg/mL 90.

**Figure 18:**
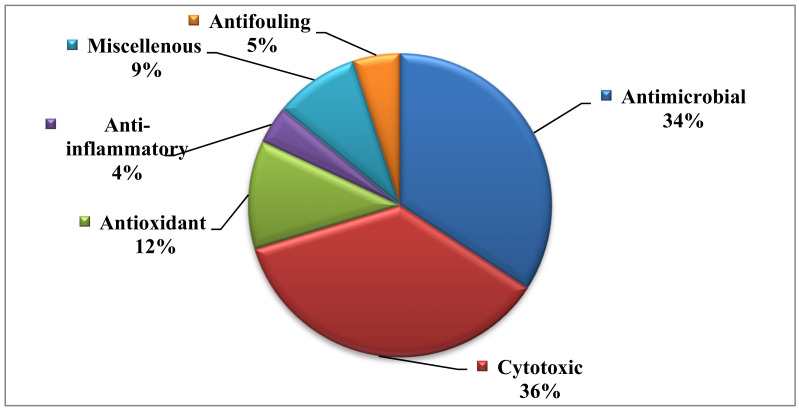
A pie chart representing the percentages of the biological activities exerted by the different bioactive alkaloids.

A pie chart representing the percentages of the biological activities exerted by the different bioactive alkaloids is represented in Figure 18. Additionally, a table summarizing the reported alkaloids, their biological activities and resources is illustrated in Table 1.

## 3. Conclusions

Thus, it can be concluded that marine-derived fungal strains are a very rich source of alkaloids. About 347 alkaloid metabolites were isolated from about twenty-six genera of fungi. About 150 alkaloids showed considerable effect with respect to the tested activities. Most of the reported bioactive alkaloids showed considerable biological activities mainly cytotoxic followed by antibacterial, antifungal, antiviral, antioxidant; however, a few showed anti-inflammatory and antifouling activities. However, the rest of the compounds showed weak or no activity toward the tested biological activities. Thus, alkaloids isolated from marine-associated fungi can afford an endless source of new drug entities that could serve as leads for drug discovery combating many human ailments. However, further investigations for additional biological activities for alkaloids that revealed no activity should be performed.

## Figures and Tables

**Table 1:** Diverse alkaloids isolated from marine-derived fungal strains and their biological activities.

Compound	Genus	Biological Activity	References
Luteoalbusin A (1) and B (2)	*Acrostalagmus*	Potent cytotoxic activity against HepG-2, MCF-7, SF-268 and NCI-H460 cell lines	[14]
Compounds (3–5)	*Acrostalagmus*		
Arthpyrones F–I (13–16)	*Arthrinium*	Notable antimicrobial potential versus *Staphylococcus aureus* and *Mycobacterium smegmatis*	[15]
Apiosporamide (19)	*Arthrinium*	Notable antimicrobial potential versus *Staphylococcus aureus* and *Mycobacterium smegmatis* revealed Observable cytotoxic activity versus U2OS and MG63	
Amauromine (21)	*Auxarthron*	Potent antagonist to CB1 receptor with a strong selective binding to human cannabinoid CB1 receptor	[16]
Chaetoglobosin E (26)	*Chaetomium*	Pronounced antiproliferative activity via induction of apoptosis on LNCaP as well as B16F10 cancer cells	[17]
ChaetoglobosinFex (28)	*Chaetomium*	Notable immunosuppressive effect Inhibition of the inflammatory mediators through toll-like receptor 4 signaling present in macrophages	[19]
N-glutarylchaetoviridin C (33)	*Chaetomium*	Significant activity versus human gastric cancer cell line (MGC-803) and human ovarian cancer cell line (HO8910)	[20]
Circumdatin A (34)	*Cladosporium*	Powerful antioxidant activity evidenced by its UV-A protection potential	[21]
(−)-Cereolactam (35)	*Coniothyrium*	Selective human leukocyte elastase inhibition	[16]
(−)-Cereoaldomine (36)	*Coniothyrium*		
Curvulamine (37)	*Curvularia*	Potent antimicrobial activity	[23]
Dichotomocej A (38)	*Dichotomomyces*	Notable activity versus human rhabdomyosarcoma cell line RD	[24]
Scequinadoline A (57)	*Dichotomomyces*	Promising inhibition to the viral production of dengue virus serotype 2	[25]
Variecolorin G (59)	*Eurotium*	Potent inhibition to Hela, Du145, SMMC7721, MCF-7, SW1990, NCI-H460 Potent antioxidant potential	[29] [34]
Isoechinulin B (60)	*Eurotium*	Potent melanogenesis inhibition using B16 melanoma cells	[32]
Neoechinulin B (61)	*Eurotium*	Notable antiviral prohibition in against influenza A/WSN/33 virus Potent antioxidant potential	[10]
Cristatumin A (62)	*Eurotium*	Powerful antibacterial activity versus *Escherichia coli*	[27]
Cristatumin B (63)	*Eurotium*	Moderate cytotoxic activity versus brine shrimp	[27]
Rubrumazines A–C (66–68)	*Eurotium*	Cytotoxic activity using brine shrimp lethal test Antibacterial activity with different degrees	[28]
Compounds (69–80)	*Eurotium*	Cytotoxic activity using brine shrimp lethal test Antibacterial activity with different degrees	[28]
12-Demethyl-12 oxoeurotechinulin B (81)	*Eurotium*	Potent activity versus Hela, Du145, SMMC7721, MCF-7, SW1990, NCI-H460	[29]
Alkaloid E-7 (85)	*Eurotium*	Potent activity versus Hela, Du145, SMMC7721, MCF-7, SW1990, NCI-H460	[29]
Dehydroechinulin (93)	*Eurotium*	Cytotoxic activity versus A-549, P-388 as well as HL-60 cell lines	[31]
Dehydrovariecolorin L (94)	*Eurotium*	Cytotoxic activity versus A-549, P-388 as well as HL-60 cell lines	[31]
Echinuline (95)	*Eurotium*	Effective melanogenesis inhibition using B16 melanoma cells Notable inhibition the barnacle larval settlement Potent antioxidant potential	[32] [33] [34]
Isoechinulin A (96)	*Eurotium*	Notable antiviral prohibition in against influenza A/WSN/33 virus Potent antioxidant potential	[10] [34]
Dihydroxyisoechinulin A (98)	*Eurotium*	Weak antifouling activity	[33]
Neoechinulin E (100)	*Eurotium*	Potent antioxidant activity	[31]
Cryptoechinuline D (101)	*Eurotium*	Potent antioxidant activity	[31]
Rubrumline D (105)	*Eurotium*	Notable antiviral prohibition in against influenza A/WSN/33 virus Effective melanogenesis inhibition using B16 melanoma cell	[10] [32]
Variecolorine O (118)	*Eurotium*	Notable antiviral prohibition in against influenza A/WSN/33 virus Potent antioxidant potential	[10] [34]
Neoechinulin C (122)	*Eurotium*	Notable antiviral prohibition in against influenza A/WSN/33 virus	[10]
Isoechinulin D (123) and C (124)	*Eurotium*	Effective melanogenesis inhibition using B16 melanoma cells	[32]
Alkaloid E-7 (125)	*Eurotium*	Highly potent melanogenesis inhibition using B16 melanoma cells	[32]
Cryptoechinulin G (126)	*Eurotium*	Effective melanogenesis inhibition using B16 melanoma cells	[32]
Eurotiumins A–B (129–130)	*Eurotium*	Moderate antioxidant potential in DPPH assay	[34]
Eurotiumins C (131) and E (133)	*Eurotium*	Highly potent antioxidant potential in DPPH assay	[34]
Eurotinoids A–C (134–136)	*Eurotium*	Potent antioxidant potential in DPPH assay	[34]
Dihydrocryptoechinulin D (137)	*Eurotium*	Potent antioxidant potential in DPPH Moderate cytotoxic effect versus SF-268 and HepG2 cell lines	[34]
Eutypellazines A–M (138–150)	*Eutypella*	Notable anti-HIV effect	[36]
Circumdatin I (151)	*Exophiala*	Potent UV-A protective behavior	[37]
Oxysporizoline (154)	*Fusarium*	Antibacterial activity versus MRSA and MDRSA Notable antioxidant potential	[38]
Tryptoquivaline T (159)	*Neosartorya*	Notable cytotoxic potential and induction of HL-60 cells apoptosis	[44]
Tryptoquivaline U (160)	*Neosartorya*	Notable cytotoxic potential and induction of HL-60 cells apoptosis	[44]
Fiscalin B (161)	*Neosartorya*	Notable cytotoxic potential and induction of HL-60 cells apoptosis	[44]
Dihydrocarneamide A (169)	*Paecilomyces*	Weak cytotoxic potential versus NCI-H460 cell lines (human large cell lung carcinoma)	[46]
Iso-notoamide B (170)	*Paecilomyces*	Weak cytotoxic potential versus NCI-H460 cell lines (human large cell lung carcinoma)	[46]
Varioxepine A (171)	*Paecilomyces*	Potent antifungal activity versus *Fusarium graminearum*	[47]
Meleagrin B (178)	*Penicillium*	Moderate cytotoxic activity inducing HL-60 cell apoptosis	[49]
Meleagrin (186)	*Penicillium*	Notable anti-proliferative potential versus HepG2 tumor cells	[68]
Brevicompanine E (189) and H (192)	*Penicillium*	Prohibition of H lipopolysaccharide (LPS)-stimulated nitric oxide formation in BV2 microglial cells	[49]
Methyl penicinoline (196)	*Penicillium*	Notable cytotoxicity versus Hep G2 cells	[51]
Penicinoline (197)	*Penicillium*	Notable cytotoxicity versus Hep G2 cells	[51]
Penitrems A, B, D, E and F (199–203)Paspaline (204)	*Penicillium*	Notable anti-migratory, anti-invasive and antiproliferative potential versus against human breast cancer cells MCF-7 cells	[52]
6-Bromopenitrem B (205) and E (206)	*Penicillium*	Notable anti-migratory, anti-invasive and antiproliferative potential versus against human breast cancer cells MCF-7 cells	[52]
Auranomides A–C (216–218)	*Penicillium*	Notable cytotoxicity versus human tumor cells	[57]
Scalusamides A–C (222–224)	*Penicillium*	Notable antibacterial and antifungal activity	[59,60,61,62]
Penicitrinine A (227)	*Penicillium*	Potent anti-proliferative activity in A-375 (human malignant melanoma	[63]
Aluminiumneohydroxyaspergillin (233)	*Penicillium*	Selective cytotoxic potential versus U937 cell line (human histiocytic lymphoma) Considerable toxicity versus brine shrimp	[65]
Communesin I (243)	*Penicillium*	Potent activity on the bradycardia induced by astemizole (ASM) in the heart rate in live zebra fish model in Potent vasculogenetic activity in vasculogenesis	[67]
Fumiquinazoline Q (244)	*Penicillium*		
Communesin A–B (245–246)	*Penicillium*		
Protuboxepin A–B (247–248)	*Penicillium*		
Prelapatin B (249)	*Penicillium*		
Glyantrypine (250)	*Penicillium*		
Variecolorins M–O (255–257)	*Penicillium*	Weak antioxidant potential in DPPH radical scavenging capacity assay	[69]
3R*,4R*dihydroxy-4-(4′-methoxyphenyl)-3,4-dihydro-2(1H)-quinolinone (260)	*Penicillium*	Cytotoxic effect on SKOV-3 cells	[70]
Shearinines D–E (268–269) and A (271)	*Penicillium*	Enhancement of apoptosis in HL-60 cells	[72]
Penipaline B and C (279–280)	*Penicillium*	Notable cytotoxic effect versus both A-549 and HCT-116 cell lines	[74]
Penipanoid A (283) and C (285)	*Penicillium*	Antimicrobial activityCytotoxic activity	[75]
Raistrickindole A (293)	*Penicillium*	Potent antiviral activity versus hepatitis C virus	[78]
Raistrickin (294)	*Penicillium*	Potent antiviral activity versus hepatitis C virus	[78]
Penicillivinacine (297)	*Penicillium*	Considerable anti-migratory potential versus the greatly metastatic MDA-MB-23 cells (human breast cancer cells)	[79]
Terretrione A (298)	*Penicillium*	Considerable anti-migratory potential versus the greatly metastatic MDA-MB-23 cells (human breast cancer cells)	[79]
Brevianamide F (300)	*Penicillium* *Pseudallescheria*	Notable antimicrobial activity versus *Staphylococcus aureus*, methicillin-resistant *S. aureus*, and multidrug-resistant *S. aureus*	[79,81]
α-Cyclopiazonic acid (301)	*Penicillium*	Antimicrobial activity against *E. coli*	[79]
Pseuboydone C (305)	*Pseudallescheria*	Cytotoxicity versus Sf9 cells	[82]
Speradine C (311)	*Pseudallescheria*	Cytotoxicity versus Sf9 cells	[82]
24,25-Dehydro-10,11-dihydro-20-hydroxyaflavinin (318)	*Pseudallescheria*	Cytotoxicity versus Sf9 cells	[82]
Scedapin C (324)	*Scedosporium*	Promising antiviral potential versus hepatitis C	[84]
Scequinadoline D (327)	*Scedosporium*	Promising antiviral potential versus hepatitis C	[84]
Scopulamide (328)	*Scopulariopsis*	Weak cytotoxic effect versus L5178Y mouse lymphoma cell line	[85]
Lumichrome (329)	*Scopulariopsis*	Weak cytotoxic effect versus L5178Y mouse lymphoma cell line	[85]
Aflaquinolone A, D, F and G (331–334)	*Scopulariopsis*	Antifouling effect against larval settlement of barnacle *Balanus amphitrite*	[86]
6-Deoxyaflaquinolone E (335)	*Scopulariopsis*	Antifouling effect against larval settlement of barnacle *Balanus amphitrite*	[86]
Didymellamide A (336)	*Stagonosporopsis*	Antifungal activity versus azole-resistant *Candida albicans*	[87]
Thielaviazoline (340)	*Thielavia*	Antimicrobial activity versus MRSA (methicillin-resistant *Staphylococcus aureus*) and MDRSA (multidrug resistant *Staphylococcus aureus)* Effective radical-scavenging activity against 2,2-dipheny1–1-picrylhydrazyl (DPPH)	[88]
Gymnastatin Z (341)	*Westerdykella*	Moderate effect versus *B. subtilis* Inhibitory potential versus MCF-7, HepG2, A549, HT-29 and SGC-7901 cell lines	[89]
Compound (343)	*Xylariaceae*	Weak antimicrobial activity	[90]
Quinolactacin A1, A2and C1 (346–348)	*Xylariaceae*	Strong antifouling potential versus *Bugula neritina* larval settlement	[90]

## Data Availability

Not applicable.
